# International Consensus Histopathological Criteria for Subtyping Idiopathic Multicentric Castleman Disease Based on Machine Learning Analysis

**DOI:** 10.1002/ajh.27743

**Published:** 2025-06-20

**Authors:** Midori Filiz Nishimura, Tomoka Haratake, Yoshito Nishimura, Asami Nishikori, Remi Sumiyoshi, Hideki Ujiie, Yuri Kawahara, Tomohiro Koga, Masao Ueki, Dorottya Laczko, Eric Oksenhendler, David C. Fajgenbaum, Frits van Rhee, Atsushi Kawakami, Yasuharu Sato

**Affiliations:** ^1^ Department of Molecular Hematopathology Okayama University Graduate School of Health Sciences Okayama Japan; ^2^ The Research Program for Intractable Disease by Ministry of Health, Labor and Welfare, Castleman Disease TAFRO and Related Ddisease Research Group Nagasaki Japan; ^3^ Division of Hematology/Oncology, Mayo Clinic Rochester Minnesota USA; ^4^ Department of General Medicine Okayama University Graduate School of Medicine, Dentistry and Pharmaceutical Sciences Okayama Japan; ^5^ Department of Immunology and Rheumatology, Division of Advanced Preventive Medical Sciences Nagasaki University Graduate School of Biomedical Sciences Nagasaki Japan; ^6^ School of Information and Data Sciences Nagasaki University Nagasaki Japan; ^7^ Department of Pathology and Laboratory Medicine Hospital of the University of Pennsylvania Philadelphia Pennsylvania USA; ^8^ Department of Clinical Immunology Hôpital Saint‐Louis Paris France; ^9^ Center for Cytokine Storm Treatment and Laboratory, Division of Translational Medicine and Human Genetics, Perelman School of Medicine University of Pennsylvania Philadelphia Pennsylvania USA; ^10^ Myeloma Center University of Arkansas for Medical Sciences Little Rock Arkansas USA

**Keywords:** clinical subtype, histopathological criteria, idiopathic multicentric castleman disease, lymphoproliferative disease, machine‐learning

## Abstract

Idiopathic multicentric Castleman disease (iMCD) is a rare lymphoproliferative disorder classified into three recognized clinical subtypes—idiopathic plasmacytic lymphadenopathy (IPL), TAFRO, and NOS. Although clinical criteria are available for subtyping, diagnostically challenging cases with overlapping histopathological features highlight the need for an improved classification system integrating clinical and histopathological findings. We aimed to develop an objective histopathological subtyping system for iMCD that closely correlates with the clinical subtypes. Excisional lymph node specimens from 94 Japanese iMCD patients (54 IPL, 28 TAFRO, 12 NOS) were analyzed for five key histopathological parameters: germinal center (GC) status, plasmacytosis, vascularity, hemosiderin deposition, and “whirlpool” vessel formation in GC. Using hierarchical clustering, we visualized subgroups and developed a machine learning‐based decision tree to differentiate the clinical subtypes and validated it in an external cohort of 12 patients with iMCD. Hierarchical cluster analysis separated the IPL and TAFRO cases into mutually exclusive clusters, whereas the NOS cases were interspersed between them. Decision tree modeling identified plasmacytosis, vascularity, and whirlpool vessel formation as key features distinguishing IPL from TAFRO, achieving 91% and 92% accuracy in the training and test sets, respectively. External validation correctly classified all IPL and TAFRO cases, confirming the reproducibility of the system. Our histopathological classification system closely aligns with the clinical subtypes, offering a more precise approach to iMCD subtyping. It may enhance diagnostic accuracy, guide clinical decision‐making for predicting treatment response in challenging cases, and improve patient selection for future research. Further validation of its versatility and clinical utility is required.

## 1 Introduction

Idiopathic multicentric Castleman disease (iMCD) is a rare lymphoproliferative disorder classified under the category of “Tumour‐like lesions with B‐cell predominance” in the 5th edition of the World Health Organization Classification of Haematolymphoid Tumours [1]. Currently, there are no established curative treatments for this disease. Many patients experience multiple relapses despite therapy, and in severe or progressive cases, the disease can be life threatening. Recent studies have indicated that iMCD comprises of multiple clinical subtypes with distinct symptoms, characteristic laboratory abnormalities, and treatment responses. Currently, three clinical subtypes have been recognized: idiopathic plasmacytic lymphadenopathy (IPL); thrombocytopenia, anasarca, fever, renal dysfunction/reticulin fibrosis, and organomegaly (TAFRO); and not otherwise specified (NOS) [2, 3, 4, 5, 6, 7, 8, 9]. Among these, the IPL and TAFRO subtypes are relatively well‐defined using the proposed clinical diagnostic criteria for each [4, 5, 6, 7, 8, 9]. Both the IPL and TAFRO subtype were originally proposed in Japan, highlighting the significant contribution of Japanese researchers to the understanding of iMCD subtypes. The IPL subtype, first proposed in Japan by Mori et al. in 1980, is characterized by hypergammaglobulinemia and generalized lymphadenopathy and generally follows a relatively indolent course with a favorable outcome [4, 7]. TAFRO syndrome was first described by Takai et al. [10] in Japan and is recognized as a unique subtype within the spectrum of iMCD [5, 11]. Diagnostic criteria for the TAFRO subtype of iMCD were subsequently established by Japanese researchers [8, 9] to better characterize and manage the TAFRO subtype, which presents with severe clinical symptoms associated with a subacute‐to‐acute course and a poor prognosis.

According to the guidelines of the Castleman Disease Collaborative Network (CDCN) [12] and the National Comprehensive Cancer Network (NCCN) [13], siltuximab is recommended as a first‐line therapy for iMCD. Tocilizumab, an IL‐6 receptor antagonist, has also been approved for the treatment of iMCD in Japan, based on an open‐label trial [14]. These anti–IL‐6 agents are the only approved therapies worldwide for iMCD; however, they generally require life‐long administration, and not all patients with iMCD respond, especially those with the TAFRO subtype [15].

**FIGURE 1:**
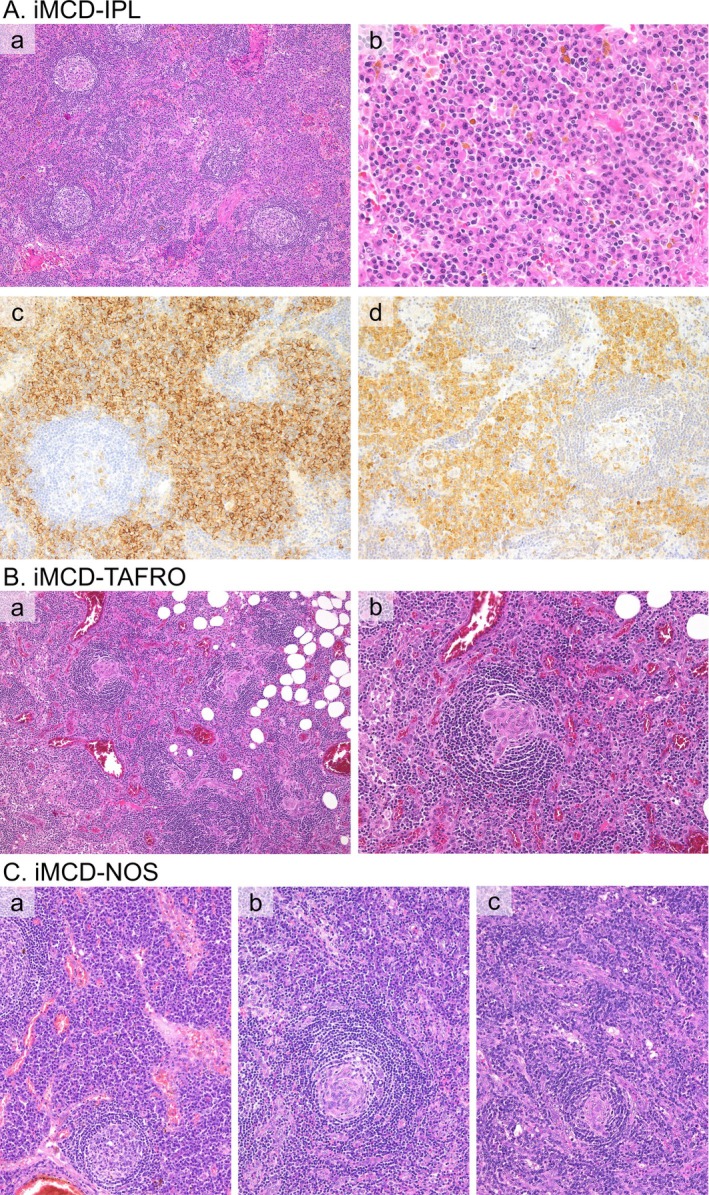
Histological Findings in each Clinical Subtype of iMCD. (A) Histological Findings in a patient with IPL subtype. (a) The germinal center is hyperplastic with a sheet‐like proliferation of mature plasma cells observed in the expanded interfollicular area (HE, 100x). (b) Magnified view of a sheet‐like proliferation of mature plasma cells. Hemosiderin deposition is observed (HE, 400x). (c) CD138‐positive plasma cells proliferate densely in a sheet‐like pattern within the interfollicular area (CD138 staining, 200x). (d) IL‐6 staining shows strong positivity in the plasma cells within the interfollicular area (IL‐6 staining, 200x). (B) Histological Findings in a patient with TAFRO subtype. (a) The germinal center is atrophic, with prominent angiogenesis both inside and outside the follicles (H&E, 100x). (b) Blood vessels enter the germinal center, forming a characteristic “whirlpool” vessel. The endothelial cells are plump. Plasma cell infiltration is not observed (HE, 200x). (C) Histological Findings in patients with NOS subtype. (a), (b), and (c) show the histological findings of different patients with NOS. (a) In the interfollicular area, a sheet‐like proliferation of mature plasma cells is observed, presenting a pattern corresponding to plasma cell‐type histology (HE, 200x). (b) Both angiogenesis and plasma cell proliferation (upper part of the field) were observed in the interfollicular area. Angiogenesis and whirlpool vessel formation were also observed within the germinal center. This pattern has been referred to as the “mixed” type, but we define it as “HyperV with plasmacytosis” (HE, 200x). (c) Prominent angiogenesis with plump endothelial cells is observed, along with blood vessels entering the germinal center and forming a “whirlpool” vessel, consistent with HyperV type histology. Plasma cell proliferation is not observed, and we define this as “HyperV without plasmacytosis” (HE, 200x). [Color figure can be viewed at wileyonlinelibrary.com]

No distinct therapeutic strategies have been defined for each clinical subtype of iMCD. However, defining the optimal patient subgroups remains a major priority [16]. Accurate diagnosis of iMCD subtype is essential for selecting the most suitable patients for particular treatments and ensuring rigorous future research. However, even for experienced hematopathologists, a definitive diagnosis of the iMCD subtype can be highly challenging [16]. Furthermore, a provisional NOS group remained for cases that did not fulfill the current diagnostic criteria for the IPL or TAFRO subtypes. Whether these NOS cases should be classified as atypical IPL or TAFRO, or maintaining them as a distinct category is justified, is yet to be clarified. Besides clinical subtyping, iMCD is histologically categorized into plasma cell (PC) and hypervascular (HyperV) types. Additionally, cases featuring both HyperV histology and abundant plasmacytosis are also present. Certain histological subtypes were frequently associated with specific clinical subtypes [17] (Figure S1). For example, IPL typically corresponds to a PC‐type histology (Figure 1A). In contrast, TAFRO usually presents with a HyperV histology (Figure 1B). The NOS subtype varies by case but typically shows a HyperV‐based histology, often presenting either “HyperV with plasmacytosis” or “HyperV without plasmacytosis”, depending on the presence or absence of plasmacytosis (Figure 1C). “HyperV with plasmacytosis” is thought to correspond to the histological pattern previously referred to as the “mixed” type. However, because of its ambiguous definition and potential for confusion, we have adopted the term “HyperV with plasmacytosis” (Figure S2). The reliability of histological patterns in determining clinical subtypes and their utility for subclassification remains uncertain.

Moreover, the boundaries among histological subtypes are not well defined, and iMCD histology is currently regarded as a continuous spectrum (Figure S1). With no universally accepted histopathological subtyping criteria, inter‐observer variability is high, reducing concordance [16].

Given that regional and individual variations in the histological subtyping of iMCD have prevented the establishment of a homogeneous disease entity for research, a robust pathological criterion to identify uniform subsets of iMCD with high diagnostic reproducibility would facilitate clinical practice and improve therapeutic strategies and patient outcomes. Thus, the establishment and validation of an objective histopathology‐based subtyping system is an urgent priority.

In the present study, we evaluated a large number of iMCD cases using predefined objective histopathological findings to develop standardized pathological criteria. We performed hierarchical cluster analysis to determine whether a homogeneous iMCD subgroup could be identified from a pathological perspective. Based on these results, we employed a machinelearning‐based decision tree classifier to develop a novel histopathological classification system aligned with iMCD clinical subtypes. This study represents a groundbreaking effort toward refining the classification framework for iMCD.

## 2 Methods

### 2.1 Study Population

We extracted 102 cases of iMCD from the lymphoma pathology consultation files of Okayama University. Five cases were excluded owing to insufficient clinical data, and three needle biopsy specimens were excluded owing to insufficient tissue samples for comprehensive histopathological evaluation. Ultimately, 94 Japanese patients (54 with IPL, 28 with TAFRO, and 12 with NOS) were included in the study. All met the current international diagnostic criteria for iMCD [17], and none were diagnosed with any autoimmune disease, infection, immunoglobulin (IgG)4‐related disease, or tumor fulfilling the exclusion criteria for iMCD. This study was approved by the Institutional Review Board of Okayama University (protocol number: 2007–033). All procedures were conducted in accordance with the principles of the Declaration of Helsinki.

### 2.2 Histopathological Evaluation

**FIGURE 2:**
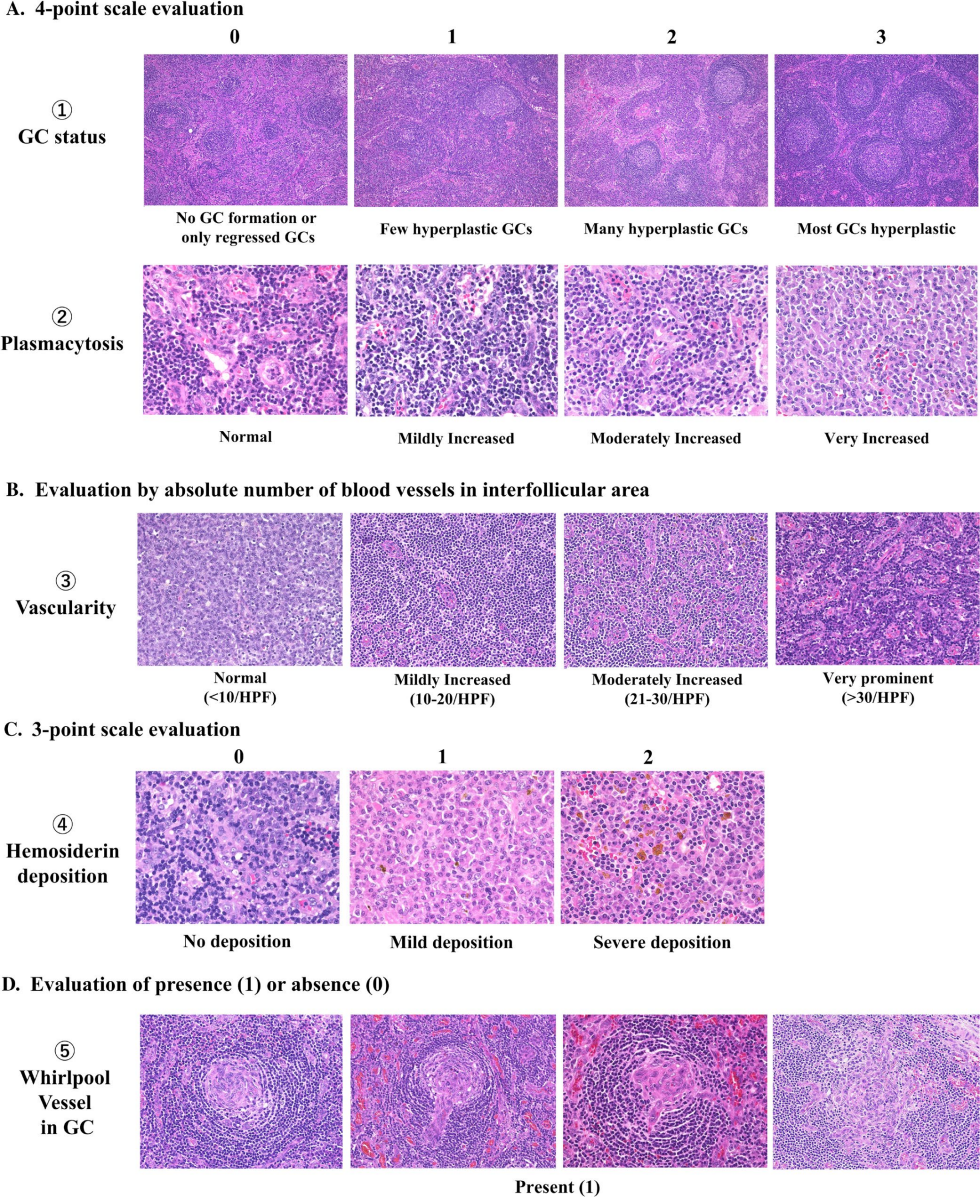
Evaluated Histological Parameters and their Scoring Criteria. Tissue evaluation was performed based on five criteria: (A) germinal center (GC) status (0–3); (B) plasmacytosis (0–3); (C) vascularity (absolute number of blood vessels per HPF in the interfollicular area); (D) hemosiderin deposition (0–2); and (E) whirlpool vessel formation in the GC (0 or 1). “Whirlpool” vessels vary and include those that form small spiral structures within the germinal center without penetrating vessels, those where vessels enter the germinal center and create a whirlpool‐like appearance at the center, and those where vessels extend radially and develop a whirlpool‐like pattern at the center of the GC. [Color figure can be viewed at wileyonlinelibrary.com]

Lymph node specimens were fixed in 10% formalin, embedded in paraffin, and sectioned (3‐μm thick) for hematoxylin and eosin (HE) staining. The following five histological parameters were evaluated (Figure 2):

- Germinal center (GC) status (scored 0–3)
- Degree of plasma cell proliferation in the interfollicular area (scored 0–3)
- Number of proliferating vessels in the interfollicular area
- Degree of hemosiderin deposition (scored 0–2)
- Presence or absence of “whirlpool vessel” in the GC (binary)

Parameters 1 and 2 were each graded on a 4‐point scale (0–3) after examining the entire lymph node. For parameter 3, the vessels were counted under three high‐power fields (HPFs) (field number: 20 mm), and the average per HPF was calculated. Hemosiderin deposition (parameter 4) was graded (0–2) using reference images. A “whirlpool vessel” (parameter 5) was defined as a blood vessel entering the GC, with endothelial cells aligning in a whirlpool‐like arrangement, suggesting directional flow [18]; it was scored as present (1) if observed anywhere in the lymph node section and absent (0) if otherwise.

Two hematopathologists (MFN and YS) independently assessed the parameters and resolved any discrepancies through consensus. All assessments were performed in a blinded manner with respect to the clinical diagnosis.

### 2.3 Treatment Response

Treatment details were assessed in patients with available medical records (IPL, 32; NOS, 5; TAFRO, 23). Tocilizumab response was retrospectively evaluated in 14 IPL and 8 TAFRO cases. Only one NOS case had both pre‐and post‐treatment data available and was therefore excluded from the response analysis. Responses were classified as “Progressive Disease”, “Partial Response”, or “Response” according to the following criteria:

#### 2.3.1 Response Criteria in iMCD‐TAFRO

- **Response**: All met:
  ○ Fluid retention improvement (pleural effusion, ascites, or generalized edema).
  ○ Platelet count ≥ 100 000/μL.
  ○ CRP < 2 mg/dL or body temperature < 37.5°C.
- **Partial Response**: At least one of the three conditions is met.
- **Progressive Disease**: None of the three conditions are met.

#### 2.3.2 Response Criteria in iMCD‐IPL

- **Response**: All met:
  ○ Lymphadenopathy improvement*.
  ○ Serum IgG < 2000 mg/dL.
  ○ CRP < 2 mg/dL or body temperature < 37.5°C.
- **Partial Response**: At least one of the three conditions is met.
- **Progressive Disease**: None of the three conditions are met.

* Improvement in lymphadenopathy was evaluated using radiologic imaging.

### 2.4 IL‐6 Immunohistochemistry and Evaluation

Immunohistochemical staining was performed using an automated Bond‐III instrument (Leica Biosystems, Wetzlar, Germany) with anti‐IL‐6 antibody (clone 10C12, 1:200; Leica Biosystems). Internal controls were used for each run. The slides were then scanned at 400× magnification using a Nanozoomer whole‐slide scanner (Hamamatsu Photonics, Hamamatsu, Japan) and analyzed using QuPath (version 0.4.3; University of Edinburgh, UK). Single‐cell detection was performed, and each cell was scored from 0 (negative) to 3 (strongly positive) based on the diaminobenzidine intensity. The H‐score was calculated after evaluating ≥ 3,000 cells in “hotspot” areas.

### 2.5 Hierarchical Cluster Analysis

We performed hierarchical cluster analysis on the 94 cases using the Ward.D2 algorithm with Euclidean distance implemented in the hclust function in R (version 4.3.2). A heatmap was generated using the pheatmap package in R (v4.3.2) to visualize the clusters.

### 2.6 Machine Learning‐Based Decision Tree Classifier

A decision tree classifier was built using the rpart package in R (v4.3.2) within the CART framework, employing the generalized Gini impurity index. Pruning was applied based on the misclassification rates. The dataset was randomly split into training (70 cases, 75%; 40 IPL, 8 NOS, and 22 TAFRO) and test (24 cases, 25%; 14 IPL, 4 NOS, and 6 TAFRO) sets for cross‐validation. The target variable was the iMCD clinical subtype (IPL, NOS, or TAFRO) and the predictors were the five histological parameters. The decision tree, which was ultimately adopted as the best‐performing model for the classification system, was evaluated not only using the accuracy score but also by visualizing the 3 × 3 precision matrix using heatmaps to assess performance across the three clinical subtypes.

### 2.7 Validation Study

For additional validation, two independent hematopathologists (MFN and YS) evaluated HE‐stained lymph node specimens from 12 iMCD cases (five IPL and seven TAFRO) provided by Nagasaki University, assessing the same five histological parameters. Assessments were performed blindly, without knowledge of the clinical subtype. The use of clinical data and pathological specimens from Nagasaki University was approved by the central ethics review board of the Nanbyo Platform operated by Kyoto University, as well as by the Institutional Review Board of Okayama University (protocol number: 2305–013).

### 2.8 Statistical Analysis

All statistical analyses were performed using R (v4.3.2). Fisher's exact test was used for categorical comparisons, and the Wilcoxon rank‐sum test was used for continuous variables. The Bonferroni correction was applied for three‐group comparisons. Statistical significance was set at *p* < 0.05.

## 3 Results

### 3.1 Demographics and Outcomes

The demographic and clinical characteristics of the 94 patients with iMCD are summarized in Table S1. No significant differences in age or sex were observed between subtypes. Regarding clinical symptoms, fever of ≥ 37.5°C and subcutaneous edema were more frequently observed in the TAFRO group than in the IPL and NOS groups (TAFRO vs. IPL: *p* < 0.001; TAFRO vs. NOS: *p* = 0.016). Pleural effusion and/or ascites were present in nearly all TAFRO (25/26, 96%) and NOS (4/4, 100%) cases, but only in 2/11 (15%) of IPL cases (IPL vs. NOS, *p* = 0.019; IPL vs. TAFRO, *p* < 0.001). No significant differences were observed among the three groups in the frequency of hepatosplenomegaly.

In laboratory findings, IPL showed elevated total protein (TP) (IPL vs. NOS, *p* = 0.005; IPL vs. TAFRO, *p* < 0.001) and IgG levels (IPL vs. NOS, *p* < 0.001; IPL vs. TAFRO, *p* < 0.001). IgG4 and other immunoglobulins were also higher in the IPL group than in the TAFRO group (*p* < 0.001), although the difference between the IPL and NOS groups was not statistically significant. Blood urea nitrogen (BUN) and serum creatinine (Cr) levels were significantly higher than in the IPL group (both *p* < 0.001). AST, ALP, and γ‐GTP levels were significantly higher in the TAFRO group than in the IPL group. Interestingly, the serum IL‐6 levels did not differ significantly among the three groups.

The H‐score for IL‐6 immunostaining was significantly lower in the TAFRO group than in the IPL and NOS groups (IPL vs. TAFRO, *p* < 0.001; NOS vs. TAFRO, *p* = 0.043).

Corticosteroids were frequently administered in all groups. Tocilizumab was used most frequently in IPL (17/32 cases, 53%), followed by TAFRO (9/23 cases, 39%) and NOS (1/5 cases, 20%). Rituximab was the second most frequently administered therapy (IPL, 13%; NOS, 20%; TAFRO, 26%). None of the patients with IPL received other immunosuppressive agents or chemotherapy. In the NOS group, mycophenolate mofetil was administered to one patient. In TAFRO, five cases were treated with cyclosporine (categorized as “Other immunosuppressants”), and three cases received chemotherapy (CyBorD, vincristine, and CVP/Bd/MP). In the IPL group (14 evaluable cases), all patients showed either a complete or partial response to tocilizumab, with 93% being fully responsive. In TAFRO (eight evaluable cases), 25% had progression and 75% had a partial response to tocilizumab; none were considered fully responsive. Patients with iMCD‐IPL showed significantly better responses to tocilizumab than those with iMCD‐TAFRO (*p* < 0.001).

### 3.2 Grading of Histological Findings

Figure S3 shows the distribution of the five histological grades across the three clinical subtypes. The GC score was significantly higher in the IPL group than in the TAFRO group (*p* < 0.001) but not in the NOS group. The NOS group had a higher GC score than the TAFRO group (*p* = 0.017). Plasmacytosis was most pronounced in IPL, significantly higher than that in both the NOS and TAFRO groups (*p* < 0.001 for each). TAFRO had the highest vascularity per HPF, followed by NOS and IPL. All pairwise comparisons were considered statistically significant (*p* < 0.001). Hemosiderin deposition was less marked, although the IPL group had significantly higher deposition than the TAFRO group (*p* < 0.001). Whirlpool vessels within the GC were observed only in NOS and TAFRO (absent in IPL), yielding significant differences between IPL and NOS (*p* = 0.002) and between IPL and TAFRO (*p* < 0.001).

### 3.3 Hierarchical Cluster Analysis

**FIGURE 3:**
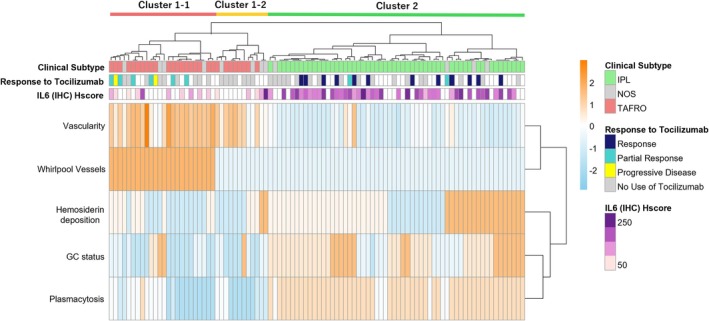
Heatmap and Hierarchical Cluster Analysis based on Histological Findings in 94 iMCD Patients. Through hierarchical cluster analysis, the cases were classified into three groups. All IPL cases grouped into the same cluster, demonstrating a highly homogeneous population. While TAFRO and IPL were never mixed within the same cluster, NOS cases were mixed within the TAFRO cluster. Additionally, the second annotation represents responsiveness to tocilizumab, and the third annotation indicates the intensity of IL‐6 immunostaining. The IPL group showed low vascularity, marked plasmacytosis, strong and wide IL‐6 expression on immunostaining, and high responsiveness to tocilizumab, which is clearly visible in the heatmap. [Color figure can be viewed at wileyonlinelibrary.com]

We performed hierarchical cluster analysis based on the five histological grades (Figure 3). In the heatmap, the histological grades ranged from low (blue) to high (orange). Each row represents a histological parameter and each column represents a patient. Cluster 1 comprised all 28 TAFRO and 8 NOS cases, whereas Cluster 2 included all 54 IPL and 4 NOS cases. Cluster 2 appeared more uniform than Cluster 1, which contained both TAFRO and NOS. Cluster 1 was further subdivided into Clusters 1–1 and 1–2 for detailed analysis. In Cluster 1, GC were generally atrophic, interfollicular vessels were markedly proliferative, and “whirlpool vessels” were more frequent. Mild plasmacytosis was present in some cases, but was less pronounced than in Cluster 2, where hemosiderin deposition was more common. Cluster 2 also showed hyperplastic germinal centers, minimal vascular proliferation, and marked plasmacytosis. No whirlpool vessels were observed in cluster 2. NOS lacks a distinct cluster, making differentiation from TAFRO challenging.

Figure 3 shows the responses to tocilizumab and IL‐6 immunohistochemical H‐scores in each cluster. In Cluster 2 (mainly IPL), patients showed better responses to tocilizumab and higher IL‐6 H‐scores, suggesting that the histologically defined clusters correlated with treatment response. NOS cases mixed within the TAFRO and IPL clusters were further investigated. Table S2 summarizes the clinical and histological characteristics of NOS cases.

Among the 12 NOS cases, 8 were grouped with TAFRO in Cluster 1 and 4 with IPL in Cluster 2. Further investigation of the clinical data of the eight patients with NOS in Cluster 1 revealed several TAFRO‐like features. For example, Case No. 2 had pleural effusion and ascites, thrombocytopenia of 5.3 × 10 [4]/μL, a high vascular score (33.3/HPF), and no plasmacytosis (score 0). Case No. 3 lacked effusions but had severe thrombocytopenia (4.5 × 10 [4]/μL) and normal IgG levels. These cases did not meet all TAFRO diagnostic criteria but showed TAFRO‐like features. Conversely, the four NOS cases in Cluster 2 had no severe thrombocytopenia and displayed elevated IgG levels in three of the four cases (though not exceeding 3500 mg/dL). One patient (Case 12) responded well to tocilizumab.

### 3.4 Decision Tree Classifiers

To build a system that classifies clinical subtypes with the highest accuracy, we developed and evaluated multiple machine learning‐based decision tree classifiers with varying maximum depths ranging from 1 to 6 (Figure S4).

Table S3 summarizes the training and test set accuracies for trees with increasing depths. Although training accuracy rises with depth, overfitting occurs at depth ≥ 4.

**FIGURE 4:**
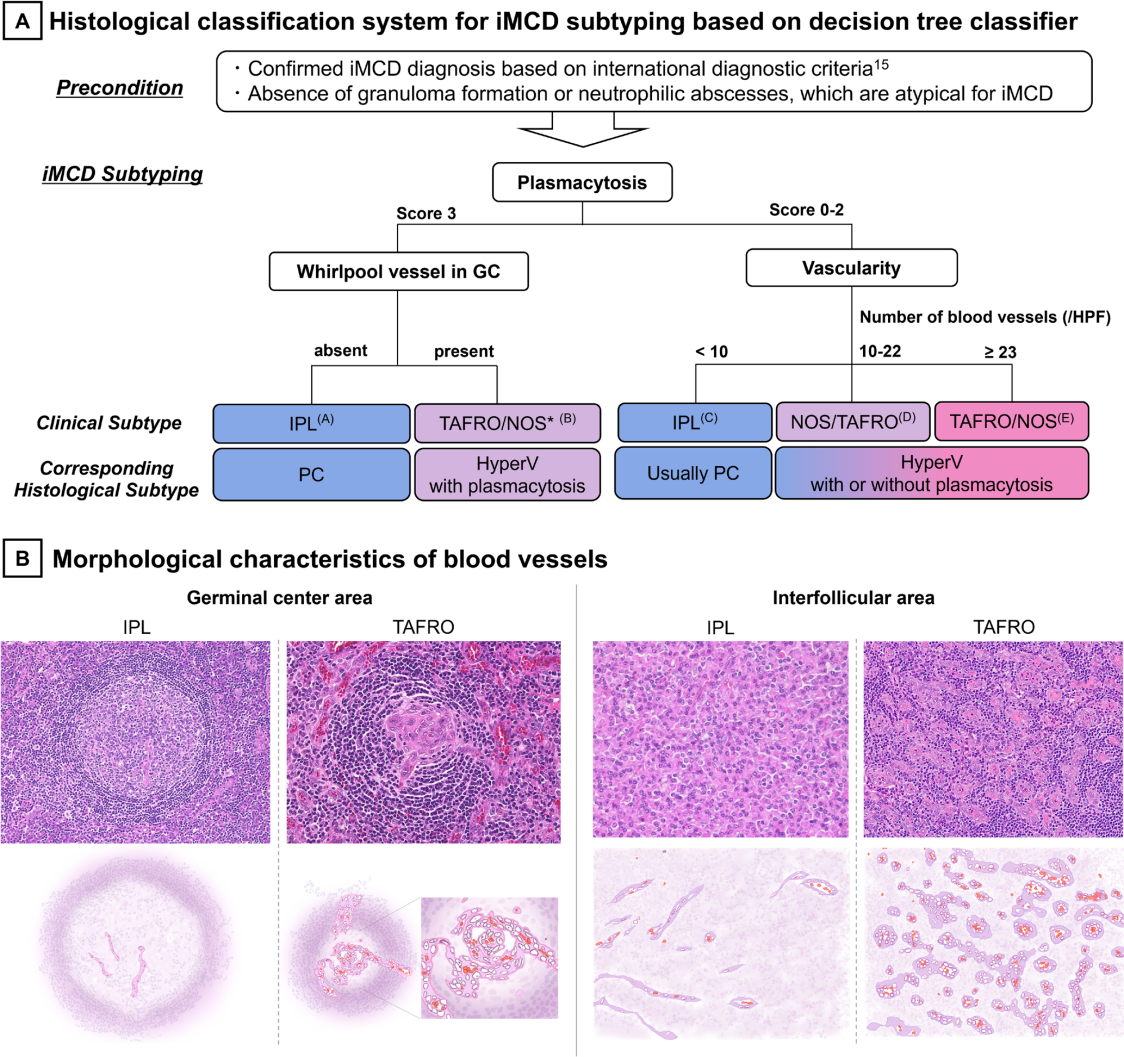
Histological Classification System for iMCD Subtyping and Key Morphological Characteristics of Blood Vessels. (A) Histological classification system for iMCD subtyping based on decision tree classifier. The application of this classification system requires patients to meet the international diagnostic criteria for iMCD [15]. A top‐to‐bottom flowchart was created. For example, there is no need to apply the whirlpool vessel criterion to individuals with a plasmacytosis score of 0–2 (although it is not problematic to apply it, the whirlpool vessel is more effectively used as an exclusion criterion for the IPL subtype). When emphasizing the correlation with clinical subtypes, PC‐type histology was defined as either the presence of score 3 plasmacytosis [Endpoint (A)] or score 2 plasmacytosis with < 10 vessels/HPF (C), both lacking whirlpool vessels. HyperV type histology is defined as lacking score 3 plasmacytosis and exhibiting ≥ 10 vessels/HPF (D, E). Rarely, HyperV may include a score of 3 plasmacytosis if vascular proliferation is prominent in whirlpool vessels (B). (B) Morphological characteristics of blood vessels (HE and schematic illustration). In the IPL subtype, vascular proliferation is minimal and the endothelium appeared flat. In contrast, the TAFRO subtype is characterized by prominent vascular proliferation, with plump endothelial cells observed in both the regressed germinal centers and interfollicular areas. Vessels penetrate the germinal centers and often exhibit a whirlpool appearance. [Color figure can be viewed at wileyonlinelibrary.com]

A decision tree with a depth of 3 was deemed the most suitable for clinical application. The precision matrices for the training and test sets are shown in Figure S5. These matrices reflect the performance of the model on the training and test data, with percentages in parentheses. Based on the decision tree, a diagnostic flowchart for clinical use was constructed (Figure 4A). From decision tree analyses, plasmacytosis, vascularity, and whirlpool vessels were identified as highly contributing parameters for subtype classification. In the classification system, in cases with a score of 3 for plasmacytosis and the presence of whirlpool vessels, the decision tree classifier classified only TAFRO. However, this may be due to an insufficient number of cases in the training data. It was considered that NOS cases could theoretically be included; therefore, in the classification system, TAFRO/NOS was listed together (*).

Our results showed that IPL and TAFRO subtypes were histologically mutually exclusive. When emphasizing the strong correlation between the IPL clinical subtypes, PC type histology should be defined as meeting either of the following criteria: the presence of score 3 plasmacytosis (corresponding to endpoint (A) in Figure 4A) or score 2 plasmacytosis with < 10 vessels/HPF (C), both lacking whirlpool vessels. Conversely, when emphasizing the correlation with the TAFRO or NOS clinical subtypes, the HyperV type histology should be defined as lacking score 3 plasmacytosis and exhibiting ≥ 10 vessels/HPF (D) and (E). In rare cases, HyperV may include a plasmacytosis score of 3 if vascular proliferation is prominent with whirlpool vessels (B).

This system is designed for subtyping iMCD patients and not for differentiating iMCD from mimicking diseases or diagnosing iMCD. This requires that patients meet the international diagnostic criteria for iMCD [17]. Epithelioid granulomas and neutrophilic abscesses are typically absent in iMCD; therefore, their presence should prompt the consideration of other diseases.

### 3.5 Validation Study

The validation results are summarized in Table S4, and their final classification using the decision‐tree system is shown in Figure S6. In all the 12 validation cases, the classification matched the actual clinical subtype (IPL or TAFRO), thereby confirming the utility and reproducibility of the proposed system.

## 4 Discussion

Our results suggest that combinations of certain histopathological parameters—particularly plasmacytosis and vascularity—are effective for identifying IPL cases. The TAFRO subtype was also accurately predicted, with TAFRO and IPL showing no overlap in any cluster, highlighting the utility of the pathological classification in predicting clinical subtypes. The NOS cases were interspersed between the IPL and TAFRO clusters, suggesting that the assessed parameters were insufficient to define NOS as a distinct histopathological entity. As illustrated in Table S2, NOS cases lacked consistent distinguishing features, although some cases were either clinically or pathologically similar to the TAFRO or IPL subtypes. Further analysis may determine whether these undefined cases share similar outcomes with the IPL or TAFRO subtypes. By leveraging the strong correlation between histopathological features and iMCD subtypes, we developed a decision tree classifier to identify key pathological parameters. Plasmacytosis was the strongest contributor, followed by vascular and whirlpool vessels. In contrast, GC status was less influential, likely due to variability within single lymph nodes. Hemosiderin deposition, reportedly influenced by IL‐6 and noted more prominently in IPL [19], also had limited discriminatory power for clinical subtyping. Follicular dendritic cell prominence has been included as a histopathological finding in the iMCD spectrum in a previous report [17]. However, we excluded FDC prominence from the evaluation because it is difficult to reliably distinguish FDCs from the vascular endothelium or pericytes in regressed germinal centers by HE staining alone, and the assessment is subject to inter‐observer variability. Although a previous study [17] assessed both regressed and hyperplastic GCs, we simplified our scoring system by focusing on GC formation as a single criterion.

Marked differences between the IPL and TAFRO subtypes were observed in the number of proliferating vessels and whirlpool vessel formation in GCs. Figure 4B highlights the morphological differences that help pathologists recognize these findings. In IPL, vascular proliferation was minimal with flat endothelial cells. In contrast, TAFRO features prominent vascular proliferation with plump endothelial cells in both the regressed GCs and interfollicular areas. Increased awareness of vascular morphology may improve diagnostic accuracy. Although the mechanism underlying whirlpool vessels is unclear, it may indicate extreme vascular proliferation. Recent study has suggested that elevated VEGFA expression in iMCD‐TAFRO could drive excessive endothelial growth and branching [18], forming these unique structures in GC.

When assessing the vascularity, fibrous proliferation around the trabeculae and blood vessels may appear in the inguinal and axillary lymph nodes. When assessing the vascularity of nodes from these sites, it is important not to diagnose such fibrosis around the vessels as HyperV type (Figure S7).

This study clarified the definitions of the PC and HyperV types, showing a stronger correlation with clinical subtypes. As shown in Figure 4A, PC‐type histology was defined by either score 3 or score 2 plasmacytosis with < 10 vessels/HPF, both without whirlpool vessels. HyperV type histology is characterized by the absence of score 3 plasmacytosis and ≥ 10 vessels/HPF, though it may include score 3 plasmacytosis in cases with prominent vascular proliferation and whirlpool vessels.

Subtyping of IPL and TAFRO follows the clinical criteria [9, 15]; however, as mimicking diseases also exist, histopathological confirmation is essential for both criteria. From a pathological perspective, IPL and TAFRO could be subtyped with sufficient accuracy. Combining clinical and pathological approaches is expected to lead to more accurate diagnoses and enhance our understanding of each iMCD subtype as a distinct disease.

In our validation cohort (Table S4), two TAFRO cases (No. 6 and No. 11) exhibited relatively modest vascular proliferation but still met the diagnostic criteria for TAFRO. Clinically, neither required intensive care, mechanical ventilation, or vasopressor support. Case No. 6 had a lower TAFRO syndrome severity score [20] than the other TAFRO cases. Although the small sample size prevents definitive conclusions, these observations suggest that lower vascular proliferation may correlate with milder clinical severity. If confirmed, this further emphasizes the importance of histopathological evaluation in clinical management.

Our classification system had certain limitations. Firstly, it is not intended for diagnosing iMCD or distinguishing it from other mimicking diseases but for subtyping confirmed iMCD cases. Therefore, it should not be applied to other lymphoproliferative neoplasms or undefined lymphadenopathies, including autoimmune and infectious diseases with overlapping histological features [21, 22, 23, 24]. Secondly, although whirlpool vessels are frequently observed in TAFRO and NOS, they may not always appear in tissue sections. Therefore, the absence of whirlpool vessels does not necessarily rule out a diagnosis of TAFRO or NOS. In contrast, no whirlpool vessels were observed in any IPL case in our cohort, highlighting their utility as a sensitive marker for excluding IPL.

As previously mentioned, this classification system is not intended for iMCD diagnosis. However, as demonstrated by our findings, it is extremely rare for IPL cases to exhibit histological features of the HyperV type, and TAFRO cases seldom show PC‐type histology. This strongly suggests that in cases with a TAFRO‐like clinical presentation but PC‐type histology, alternative causes, such as autoimmune diseases, should be considered.

Despite these, our classification system was designed to enable pathologists with limited experience in Castleman disease to perform a reliable evaluation using routine staining, while correlating well with clinical subtypes. This study provides the first pathology‐driven evidence that IPL represents a highly uniform disease cluster, characterized by high IL‐6 immunohistochemical H‐scores [A.N., M.F.N., and Y.S., manuscript submitted March 2025] and a favorable response to tocilizumab. Beyond its diagnostic utility, these findings suggest that iMCD subtyping based on this system can inform subtype‐specific treatment strategies, and ultimately improve patient outcomes. Additionally, by reducing interobserver variability, this system may minimize misclassification in future iMCD research, facilitating consistent interpretation and integration of data across different investigative groups.

Considering that our study primarily involved Asian patients, validation in diverse global cohorts is essential to establish an international standard. In the future, the incorporation of digital pathology techniques could enhance the standardization and reproducibility of classification systems. Additionally, deeper biological characterization through comprehensive cytokine profiling and angiogenesis factor analysis could offer further insights into the mechanistic differences between subtypes.

## Author Contributions

Y.S. and M.F.N. designed the research. Data collection and analysis were performed by M.F.N., T.H., A.N., H.U., Y.N., Y.K., and R.S. Pathological evaluations were conducted by M.F.N., Y.S., and A.N. Data visualization and illustration creation were contributed by T.H., Y.K., and M.F.N. Machine learning analysis was performed by M.F.N. and M.U. The manuscript was written by M.F.N. Y.S., A.K., T.K., Y.N., D.C.F., F.v.R., E.O., and D.L. supervised the study and edited the manuscript. All authors have reviewed and approved the final manuscript.

## Ethics Statement

This study was approved by the Institutional Review Board of Okayama University (protocol number: 2007–033). The use of clinical data and pathological specimens from Nagasaki University was approved by the central ethics review board of the Nanbyo Platform operated by Kyoto University, as well as by the Institutional Review Board of Okayama University (protocol number: 2305–013). All procedures complied with the Declaration of Helsinki.

## Conflicts of Interest

E.O. has served as a consultant for Recordati Rare Diseases and as an expert on the advisory board for CSL Behring. D.C.F. has received research funding and consulting fees from Recordati Rare Diseases. The remaining authors declare no competing financial interests. F.v.R. has served on advisory boards for Adicet Bio, Bristol Myers Squibb, Castleman Disease Collaborative Network, EUSA Pharma, GlaxoSmithKline, Janssen Pharmaceuticals Inc., Kite Pharma, Secura Bio, Myeloma360, and Recordati. Additionally, F.v.R. has received donated study drugs for clinical trials from Pfizer.

## Supporting information


Data S1.

## Data Availability

The data that support the findings of this study are available from Dr. Midori Filiz Nishimura upon reasonable request (midorifiliz-nihimura@okayama-u.ac.jp).
