# Supplementary material for: International Consensus Histopathological Criteria for Subtyping Idiopathic Multicentric Castleman Disease Based on Machine Learning Analysis
Source: Am J Hematol. 2025 Jun 20;100(9):1502–12. doi: 10.1002/ajh.27743 (PMC12326216; doi:10.1002/ajh.27743)
Supplement: Supplementary file 1 — Data S1. [file AJH-100-1502-s001.pdf]

Supplemental Tables

Supplemental Table 1.
The Demographic and Clinical Characteristics of 94 iMCD patients

	Clinical subtype			*p*		
	① IPL n=54	② NOS n=12	③ TAFRO n=28	①vs.②	①vs.③	②vs.③
**Age (median, range)**	54 (29-76)	51.5 (32-70)	50.5 (25-79)	1	1	1
**Gender (male: female)**	30:24	6:6	19:9	1	1	0.933
**Symptoms (n/N, %)**						
Fever(≧37.5℃)	9/25 (36)	5/9 (56)	20/20 (100)	1	**< 0.001****	**0.016***
Pleural effusion / Ascites	2/11 (15)	4/4 (100)	25/26 (96)	**0.019****	**< 0.001****	1
Subcutaneous edema	9/25 (36)	5/9 (56)	20/20 (100)	1	**< 0.001****	**0.016***
Hepatomegaly/Splenomegaly	10/20 (50)	4/5 (80)	11/15 (73)	1	0.887	1
**Laboratory findings (median, range)**						
Hb (g/dL)	10.1 (4.2-14.2) [n=52]	10.1 (6.8-13.3) [n=10]	9.4 (6.3-14.0) [n=26]	—	—	—
Male	10.7(7-14.2)	11.8(9.4-13.3)	9.2(6.8-14)	1	0.36	0.3
Female	9.75(4.2-12.5)	8(6.8-10.9)	10.8(6.3-12.7)	1	0.61	0.44
Plt (×10^3/μL)	389 (134-424) [n=52]	238 (45-788) [n=12]	3.8 (4.0-99) [n=27]	0.065	**< 0.001****	**< 0.001****
TP (g/dL)	9.9 (6.9-14.8) [n=36]	8.1 (6.0-10.3) [n=10]	5.8 (3.8-7.9) [n=19]	**0.005***	**< 0.001****	**< 0.001****
Alb (g/dL)	2.6 (1.8-3.6) [n=30]	2.9 (1.3-3.9) [n=10]	2.1 (1.1-3.5) [n=25]	1	**0.015***	0.145
BUN (mg/dL)	13.5 (6.0-38.0) [n=23]	14.9 (9.5-61.1) [n=8]	46.0 (13.8-95.0) [n=14]	1	**< 0.001****	0.059
Cre (mg/dL)	0.75 (0.46-4.02) [n=39]	0.94 (0.57-4.42) [n=10]	1.13 (0.40-39.1) [n=24]	—	—	—
Male	0.875(0.66-1.6)	1.43(0.81-4.42)	1.08(0.74-17.3)	0.215	**0.014***	1
Female	0.68(0.46-4.02)	0.68(0.57-1.02)	1.18(39.1-0.4)	0.86	0.09	0.61
CRP (mg/dL)	6.25 (0.72-22.4) [n=52]	7.26 (1.19-19.4) [n=11]	16.1 (1.65-29.4) [n=27]	1	**< 0.001****	0.113
IgG (mg/dL)	4753 (2872-9347) [n=53]	3165 (1479-4371) [n=11]	1360 (867-2512) [n=22]	**< 0.001****	**< 0.001****	**< 0.001****
IgG4 (mg/dL)	743(161-3360) [n=25]	66.6 (11.4-2824) [n=5]	34.7 (18.0-135) [n=7]	0.223	**< 0.001****	1
IgA (mg/dL)	537 (95-1487) [n=42]	507 (166-1076) [n=10]	207 (137-363) [n=20]	1	**< 0.001****	**< 0.001****
IgM (mg/dL)	210 (53-427) [n=41]	170 (34.7-643) [n=10]	65.25 (37.0-137) [n=20]	0.677	**< 0.001****	**0.033***
IL-6 (pg/mL)	28.6 (14.5-100) [n=12]	20.8 (6.34-275) [n=7]	20.2 (8.8-579) [n=17]	0.897	0.849	1
Ferritin (ng/mL)	132 (25.6-324) [n=13]	269 (26-1268) [n=8]	605 (15-2202) [n=15]	0.267	**< 0.001****	0.299
VEGF (pg/mL)	558 [n=1]	1510 [n=1]	237 (90-627) [n=7]	NA	NA	NA
PT (sec)	13.2 (10.9-86.4) [n=12]	13.1 (11.1-31.6) [n=7]	13.5 (10.7-16.8) [n=10]	1	1	1
APTT (sec)	36.1 (27.6-48.9) [n=15]	40.6 (33.1-55.8) [n=8]	34.1 (25.9-121) [n=13]	0.388	1	1
FDP (μg/mL)	3.3 (3.1-3.9) [n=3]	10 (3.0-25.7) [n=4]	20.9 (6.5-92.9) [n=15]	1	**0.007***	0.24
D-dimer (μg/mL)	0.80 (0-23.7) [n=10]	5.3 (1.5-11.6) [n=5]	13.2 (1.1-86.6) [n=15]	0.07	**0.002***	0.449
**IL-6 immunostaining (H score) (median, range)**	153 (53-268) [n=40]	214 (70-262) [n=3]	33 (1.1-190) [n=13]	1	**<0.001****	**0.043***
**Treatment (n/N, %)**						
Corticosteroid	24/32 (75)	4/5 (80)	23/23 (100)	—	—	—
Tocilizumab	17/32 (53)	1/5 (20)	9/23 (39)	—	—	—
Rituximab	4/32 (13)	1/5 (20)	6/23 (26)	—	—	—
Other immunosuppressants	0/32 (0)	1/5 (20)	5/23 (22)	—	—	—
Chemotherapy	0/32 (0)	0/5 (0)	3/23 (13)	—	—	—
**Response to tocilizumab (n/N, %)**						
Unresponsive	0/14 (0)	NA	2/8 (25)	NA	**< 0.001****	NA
Partially responsive	1/14 (7)	NA	6/8 (75)			
Responsive	13/14 (93)	NA	0/8 (0)			

N indicates the number of the population. Abbreviations: Hb, Hemoglobin; PLT, Platelet; TP, Total protein, Alb, Albumin; BUN, Blood Urea Nitrogen; Cre, Creatinine; CRP, C-reactive protein; IL-6, Interleukin-6; PT, Prothrombin Time; APTT, Activated partial thromboplastin time; FDP, Fibrinogen/fibrin degradation products; NA, Not available. Bold values indicate statistically significant results ($p < 0.05$).

Supplemental Table 2. Clinical and Pathological Findings in All NOS Cases

Case No.	Cluster	Scores of Histological parameters					Symptom		Laboratory findings			IL6 (IHC) H score	Response to TCZ
		GC	Vascu -larity	Whirlpool vessel	Plasma-cytosis	Hemosiderin deposition	Pleural effusion /Ascites	Plt (x10^4/ µL)	IgG (mg/dL)	CRP (mg/dL)	Cre (mg/dL)		
1	1	1	20.3	0	1	2	NA	27.7	3783	8.89	NA	261.5	NA
2	1	1	33.3	1	0	0	Present	5.3	NA	16.1	1.02	NA	NA
3	1	0	17.3	0	0	1	Absent	4.5	1479	12.5	0.68	NA	No TCZ
4	1	0	21.0	1	1	1	Absent	18.3	2550	19.4	4.42	NA	No TCZ
5	1	1	21.7	0	2	0	Absent	35.8	3348	4.1	NA	NA	No TCZ
6	1	3	28.0	1	2	1	Absent	19.8	4372	6	1.94	NA	No TCZ
7	1	1	19.3	0	1	2	Absent	36.6	3165	3.21	0.6	69.7	Unresponsive
8	1	3	15.0	1	2	0	NA	NA	NA	NA	NA	NA	NA
9	2	2	7.0	0	3	2	NA	42.1	3269	1.2	0.81	214.3	NA
10	2	2	13.7	0	2	1	Absent	78.8	2576	14.5	0.57	NA	No TCZ
11	2	2	8.7	0	3	2	Present	17.4	1723	1.5	1.43	NA	NA
12	2	1	18.7	0	2	2	Absent	40.4	2848	7.26	0.98	NA	Responsive

Abbreviations: GC, Germinal center, PLT, Platelet; IgG, Immunoglobulin G; CRP, C-reactive protein; Cre, Creatinine; NA, Not available; IHC, Immunohistochemistry; TCZ, Tocilizumab.

Supplemental Table 3. Accuracy of Decision Tree Models on Training and Test Sets by Tree Depth

Maximum depth of the tree	Accuracy (%)	
	training set	test set
1	84.3	70.8
2	88.6	83.3
3	91.4	91.7
4	92.9	87.5
5	95.7	79.2
6	94.3	70.8

Supplemental Table 4. Pathological and Clinical Characteristics of These Validation Cases

No.	Age	Sex	Clinical subtype	Clinical subtype according to a novel classification system	GC status (0—3)	Vascularity (/HPF)	Whirlpool vessel	Plasmacytosis (0—3)	Hemosiderin deposition (0—2)	Plt ($\times 10^4/\mu L$)	Hb (g/dL)	CRP (mg/dL)	Cre (mg/dL)	IgG (mg/dL)	Pleural effusion /Ascites	HD	PS	TAFRO syndrome severity score	ICU management	NPPV/NHF	Use of vasopressor drug	Treatment
1	66	M	IPL	IPL	2	6	Absent	3	1	338000	11	4.7	0.94	4436	0	0	0	—	No	No	No	TCZ
2	33	F	IPL	IPL	3	3.3	Absent	3	3	324000	11.1	2.69	0.54	4122	0	0	0	—	No	No	No	TCZ
3	43	F	IPL	IPL	3	4.7	Absent	3	3	456000	9.3	3.7	0.49	3733	0	0	0	—	No	No	No	TCZ
4	50	F	IPL	IPL	2	3.7	Absent	3	3	424000	9	7.22	0.68	4521	0	0	1	—	No	No	No	TCZ
5	62	M	IPL	IPL	0	7.7	Absent	2	2	262000	8.5	6.72	1.98	4088	0	0	0	—	No	No	No	TCZ
6	47	M	TAFRO	NOS (/TAFRO)	0	10	Absent	2	0	63000	6.4	14.25	7.13	5833	1	1	3	8	No	No	No	TCZ
7	47	F	TAFRO	TAFRO	0	34.3	Present	0	0	80000	8	21.50	1.72	1931	1	1	3	11	No	No	No	PSL+TCZ→TCZ
8	46	M	TAFRO	TAFRO	0	40.3	Present	0	0	82000	13.6	27.84	3.28	1094	1	1	3	10	No	No	No	PSL+TCZ+sirolimus→TCZ
9	66	M	TAFRO	TAFRO	1	30	Present	2	2	40000	10.3	23.77	1.34	1070	1	0	3	10	Yes	NPPV	No	PSL+TCZ→RTX→TCZ →RTX→sirolimus
10	64	M	TAFRO	TAFRO	0	46.3	Present	0	0	24000	9.7	12.69	2.59	862	1	1	3	10	Yes	NHF	NAd	PSL+TCZ+sirolimus→TCZ
11	63	M	TAFRO	NOS (/TAFRO)	0	15.7	Present	1	1	11000	8.4	29.15	2.82	1348	1	1	2	11	No	No	No	mPSL→TCZ→PSL→filgontinib
12	52	F	TAFRO	TAFRO	0	35	Present	0	0	44000	9	24.88	1.46	1112	1	1	3	11	Yes	NHF	NAd	PSL+TCZ

Abbreviations: GC, Germinal center; HPF, High-power field; Plt, Platelet; Hb, Hemoglobin; CRP, C-reactive protein; Cre, Creatinine; IgG, Immunoglobulin; HD, Hemodialysis; PS, Performance status; ICU, Intensive care unit; NPPV, Noninvasive Positive Pressure Ventilation; NHF, Nasal high flow; TCZ, Tocilizumab; PSL, Prednisolone; RTX, Retuximab

Supplemental Figures

Supplemental Figure 1.

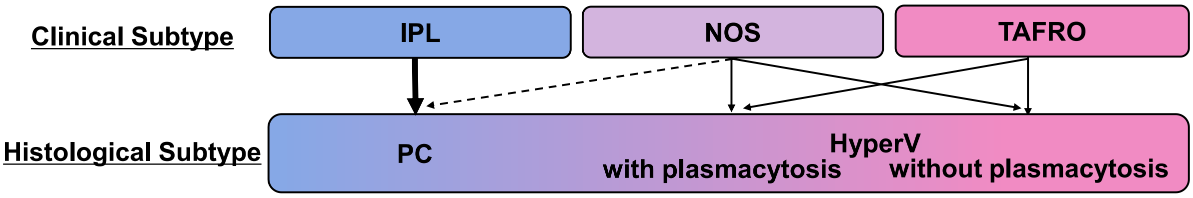

Predominant Findings Observed in each Histological Subtype

GC	Hyperplastic	Atrophic	Atrophic
Plasmacytosis	Prominent	Moderate	Minimal
Vascularity	Mild	Moderate ~ Severe	Severe
"Whirlpool vessel"(◄)	Absent	Sometimes	Often

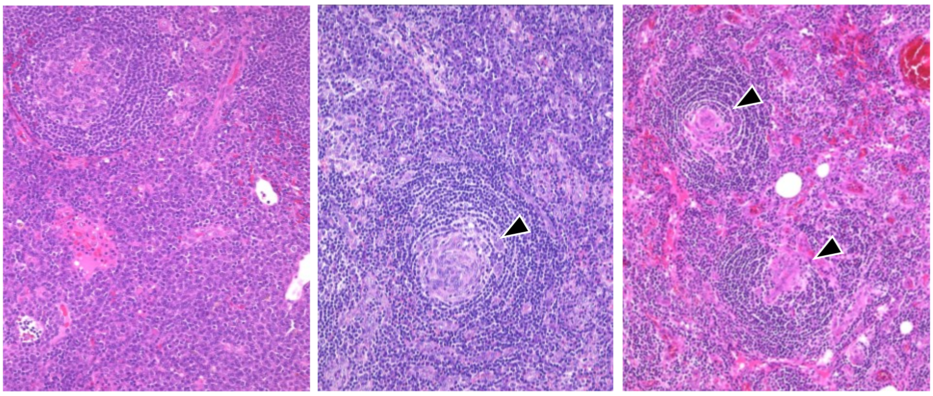

Correlation Between Clinical and Histological Subtypes of iMCD

Each clinical subtype exhibits some degree of association with specific histological subtypes. For example, IPL typically corresponds to plasma cell(PC)-type histology. TAFRO subtype usually presents hyper-vascular (HyperV) histology, occasionally showing a HyperV with plasmacytosis histology. NOS subtype demonstrates varying levels of plasmacytosis and vascular proliferation. The boundaries between each histological subtype are not well defined, and the histological features of iMCD are currently regarded as a continuous spectrum.

Supplemental Figure 2.

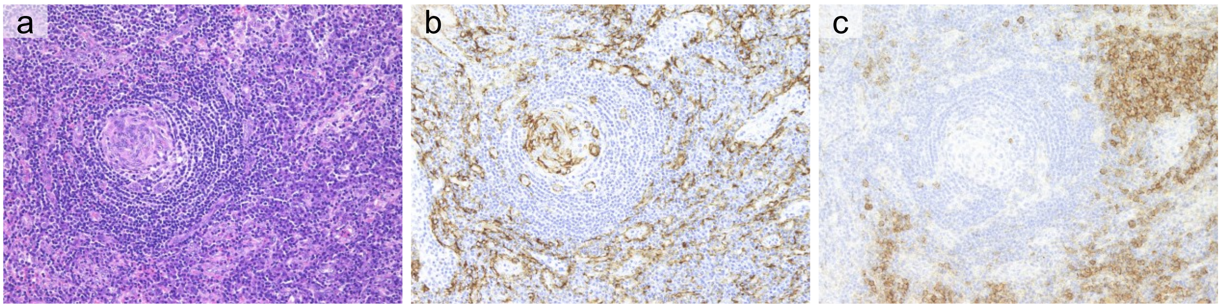

Histological Pattern Corresponding to HyperV with Plasmacytosis

(a) A histological pattern showing both vascular proliferation and plasmacytosis, with the presence of whirlpool vessels in the germinal center (HE, 200x).

(b) Immunostaining for αSMA reveals positive staining in the smooth muscle of the vascular walls, indicating a hyper-vascular lesion (αSMA, 200x).

(c) Infiltration of CD138-positive plasma cells is also observed (CD138, 200x).

Supplemental Figure 3.

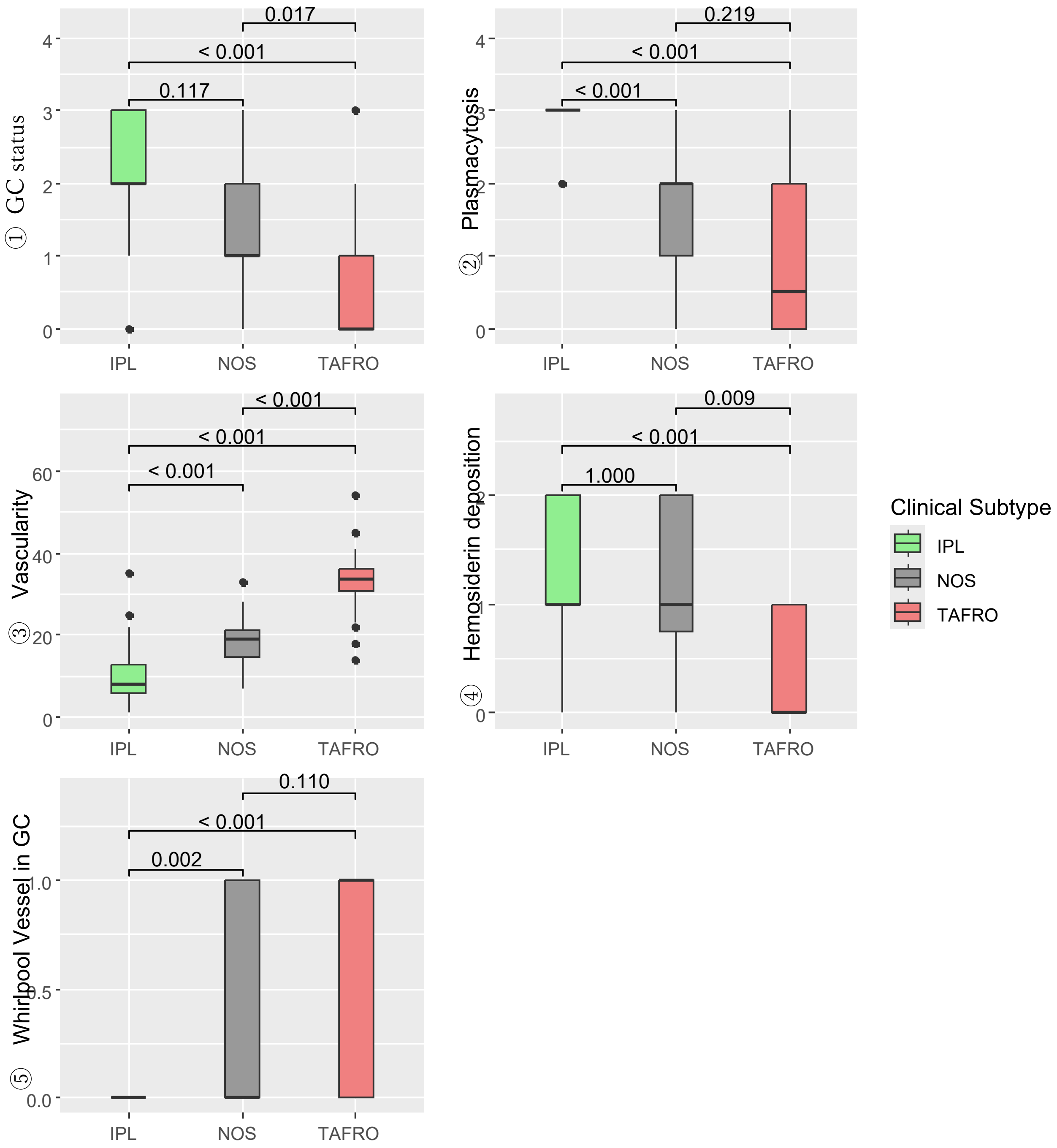

Comparison of Histological Grades Among Clinical Subtypes

Data are shown as boxplots representing the median (line) and quartiles (25th and 75th percentile) with whiskers extending ±1.5×interquartile range.

Supplemental Figure 4.

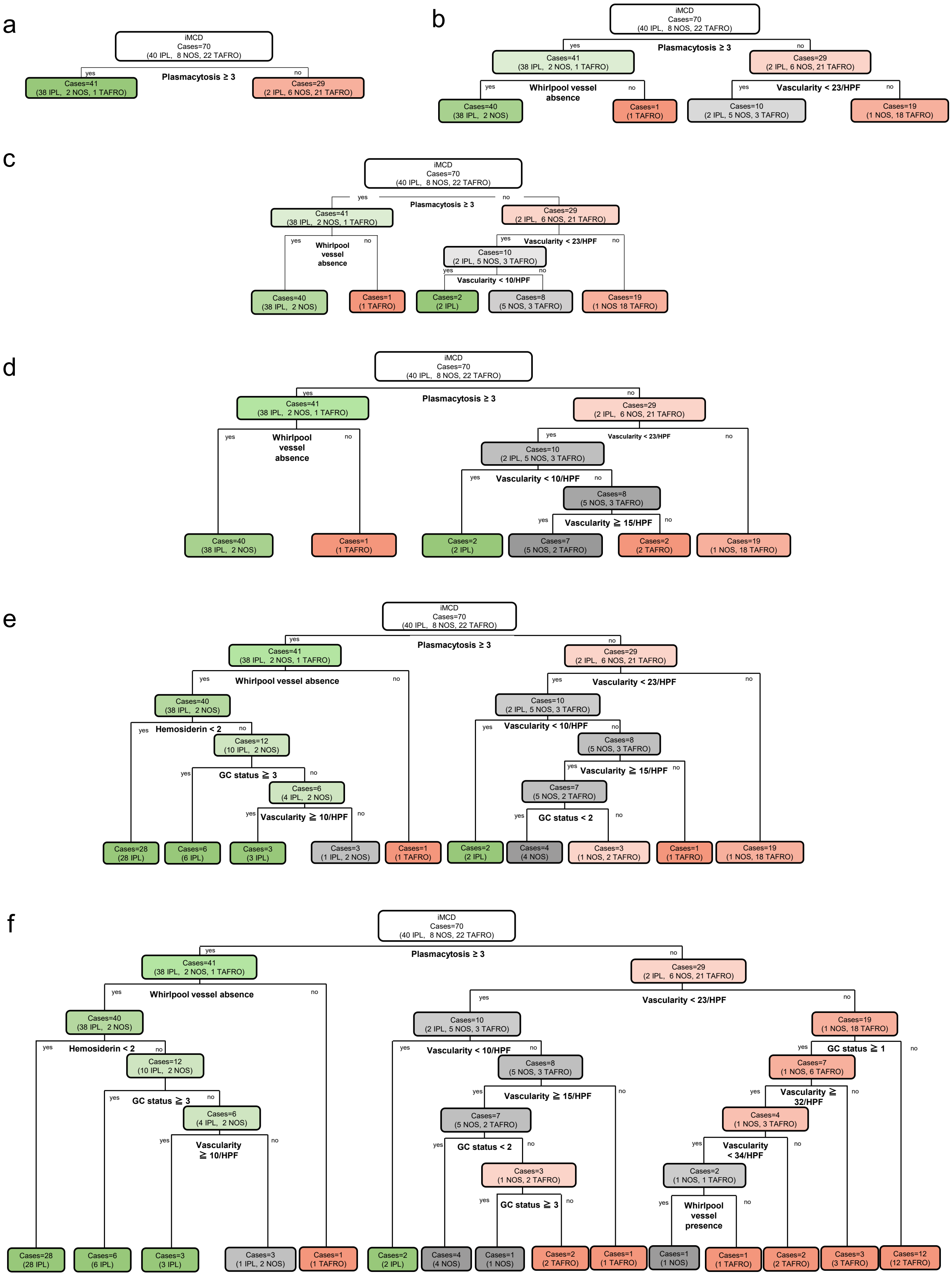

Decision Trees for the Diagnosis of Clinical Subtypes of iMCD, Obtained from the Training Data

The color of nodes indicates the clinical subtype: green = IPL, orange = TAFRO, gray = NOS. The darker color indicates a higher proportion of each disease. (a) to (f) represent decision trees with maximum depths ranging from 1 to 6.

(a) In a single-node tree (maximum depth = 1), plasmacytosis was the strongest discriminator between IPL and TAFRO. Among the 70 training cases, 41 had a plasmacytosis score of 3 (38 IPL, 2 NOS, and 1 TAFRO), whereas 29 had a score <3 (2 IPL, 6 NOS, and 21 TAFRO). The training accuracy was 84% (59/70), and the test accuracy in the 24-case validation set was 71% (17/24).

(b) A tree with a maximum depth of 2 (three decision nodes) classified cases based on plasmacytosis, whirlpool vessel, and vascularity. Among the cases with a plasmacytosis score of 3, the presence of a whirlpool vessel predicted TAFRO. In those with plasmacytosis <3, vascularity ≥23/HPF differentiated TAFRO/NOS from IPL/NOS. The training and test accuracies were 89% (62/70) and 83% (20/24), respectively.

(c) A tree with a maximum depth of 3 (four decision nodes) achieved training and test accuracies of 91% and 92%, respectively.

(d) A tree with a maximum depth of 4 (five decision nodes) yielded 93% training accuracy and 88% test accuracy.

(e) A tree with a maximum depth of 5 (nine decision nodes) showed improved training accuracy (96%), but the test accuracy declined to 80%, suggesting potential overfitting.

(f) A tree with a maximum depth of 6 (14 decision nodes) attained 94% training accuracy and 71% test accuracy, indicating that increased model complexity did not enhance generalizability.

Supplemental Figure 5.

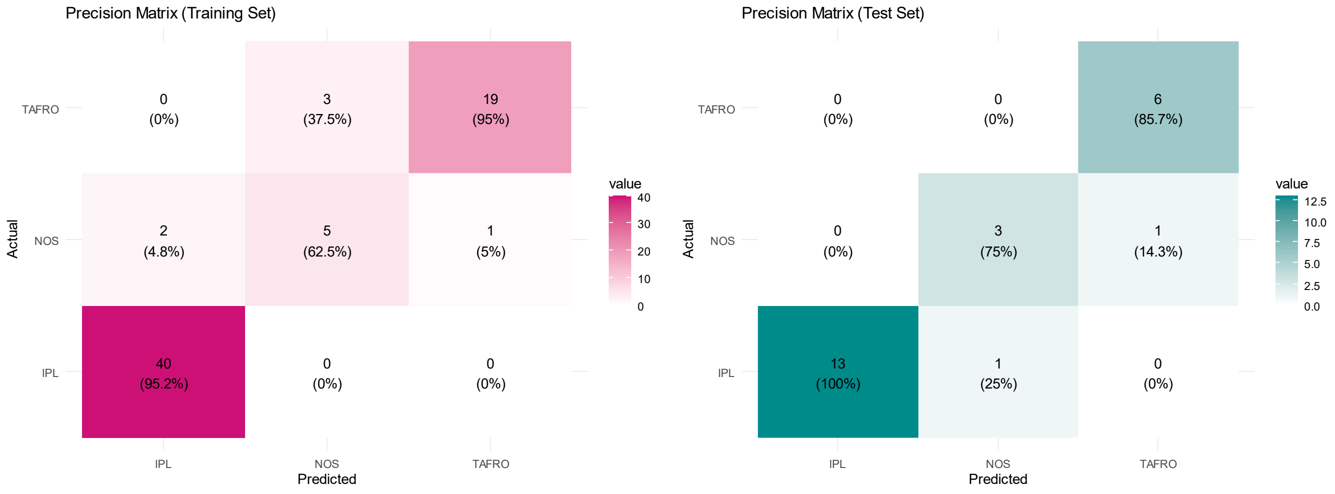

Precision Matrices for the Decision Tree Classifier (maximum depth = 3) Applied to the Training and Test-sets

The matrices show the number of correctly and incorrectly classified instances for each class, with percentages provided in parentheses. The training-set matrix reflects the model's in-sample performance, while the test set matrix shows the out-of-sample performance, highlighting the generalization ability of the model.

Supplemental Figure 6.

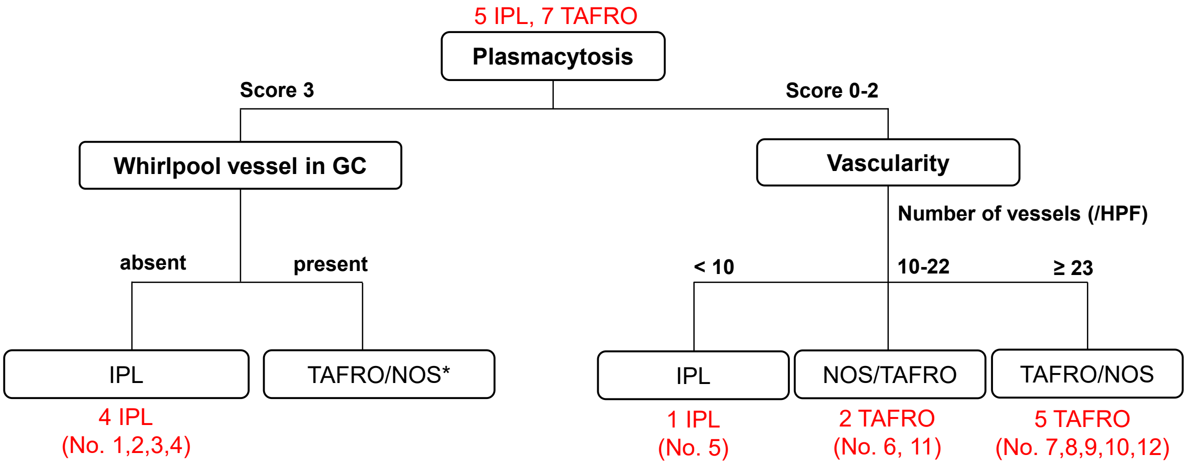

Results of Applying the Classification System to the Validation Cohort

Supplemental Figure 7.

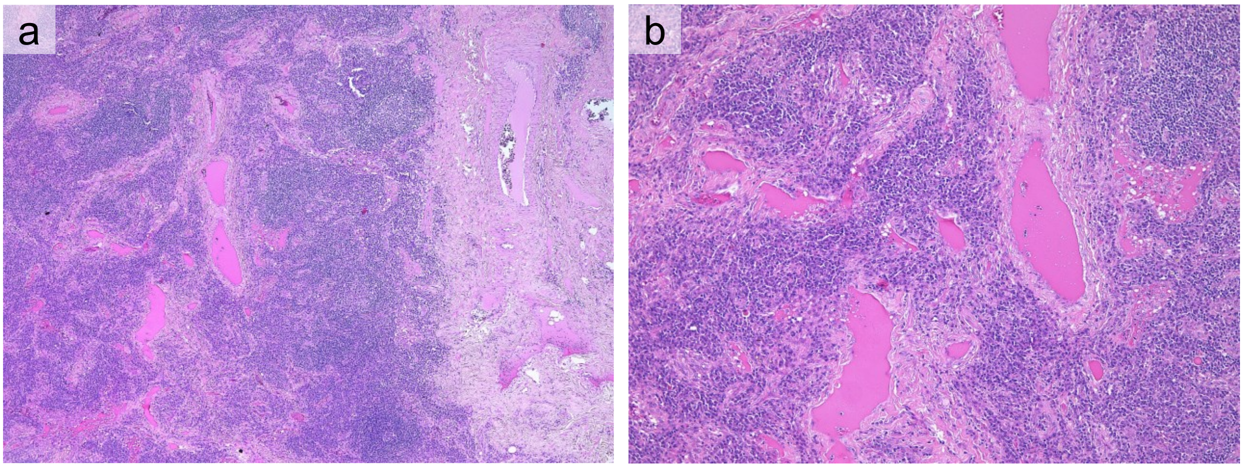

Histological Findings of Inguinal Lymph Nodes in iMCD-IPL Patients

(a) Prominent fibrous proliferation around the lymph node trabeculae and blood vessels may give the impression of abundant vascular proliferation, requiring careful attention. (b) It is important not to diagnose it as the hyper-vascular type histology based on the existing vascular structures.
